# Development of a Portable Gait Rehabilitation System for Home-Visit Rehabilitation

**DOI:** 10.1155/2015/849831

**Published:** 2015-04-05

**Authors:** Hiroaki Yano, Naoki Tanaka, Kiyotaka Kamibayashi, Hideyuki Saitou, Hiroo Iwata

**Affiliations:** ^1^University of Tsukuba, 1-1-1 Tennodai, Tsukuba 305-8573, Japan; ^2^Tsukuba Memorial Hospital, 1187-299 Kaname, Tsukuba 300-2622, Japan; ^3^Doshisha University, 1-3 Tataramiyakodani, Kyotanabe, Kyoto 610-0394, Japan; ^4^Tsukuba Memorial Hospital, 1-1-1 Tennodai, Tsukuba 305-8573, Japan

## Abstract

This paper describes the development of a gait rehabilitation system with a locomotion interface (LI) for home-visit rehabilitation. For this purpose, the LI should be compact, small, and easy to move. The LI has two 2 degree-of-freedom (DOF) manipulators with footpads to move each foot along a trajectory. When the user stands on the footpads, the system can move his or her feet while the body remains stationary. The footpads can have various trajectories, which are prerecordings of the movements of healthy individuals walking on plane surfaces or slopes. The homes of stroke patients may have not only flat surfaces but also some slopes and staircases. The quadriceps femoris muscle is important for walking up and down slopes and staircases, and the eccentric and concentric contractions of this muscle are, in particular, difficult to train under normal circumstances. Therefore, we developed a graded-walking program for the system used in this study. Using this system, the user can undergo gait rehabilitation in their home, during visits by a physical therapist. An evaluation of the results of tests showed that the vastus medialis muscles of all the subjects were stimulated more than by walking on real slopes.

## 1. Introduction

In recent years, in many countries, rehabilitation at home with a visiting physical therapist has become more widespread. The effectiveness of home-visit rehabilitation has been confirmed in several research studies [1, 2]. In Japan, because of the reduction in hospital treatment, the importance of home-visit rehabilitation has increased. The number of patients who undergo home-visit rehabilitation is increasing in Japan. About 0.12 million patients received home-visit rehabilitation in 2012 [3]. In gait rehabilitation for poststroke patients, the typical training during a home-visit involves outdoor walking around the patient's home with walking aids such as a cane. The exercise is chosen by considering the patient's condition, the restricted time frame for rehabilitation, and the therapist's workload. However, the amount of exercise that the patient can do in the short time available is insufficient for him or her to maintain or develop their walking ability.

To address this problem, many robotic assisted systems have been developed. Although they showed effectiveness for rehabilitation, most of them are used in hospitals. For home use, the system needs to be compact and small. That might, however, cause some degradation in the performance of the system such as a reduction in the rated walking speed. In addition, to reduce the risk of falling, the strength of the vastus medialis muscle plays an important role [4]. In particular, eccentric and concentric contractions of this muscle are required when the user descends or climbs a slope or staircase. It is difficult, however, for the patient to strengthen or maintain the activity of this muscle in normal circumstances. If the system can display a virtual slope or staircase, the user can train his or her vastus medialis muscle while walking with a natural motion. To present a virtual staircase, the robotic system has to have sufficient power to display the staircase and, also, to stop the footpads at the correct positions for simulation [5]. In addition, pressure sensors to detect the shift of the weight on the footpads are required. These features lead to increases in the cost of development.

The objectives of this study, therefore, were to design and develop a small and compact gait rehabilitation system, and develop a method with which the vastus medialis muscle of a patient could be trained using this system. The system developed in this study is small and can be used to train the concentric and eccentric contractions of the vastus medialis muscle, which are needed for a person to climb or descend a slope or a staircase. The effectiveness of this system was verified through experiment.

## 2. Related Works

Recently, many robotic assisted systems have been developed. For upper limbs, there are many robotic systems for home-visit rehabilitation [6, 7], and their effectiveness has been verified through experiments. On the other hand, for lower limbs, some rehabilitation systems using robotics have been developed for use in hospitals. There are 3 types of robotic systems for gait rehabilitation. One is the treadmill type system that moves backwards as the user walks on it. Another is the exoskeletal type system, which is attached to the user's body to support flexion and extension of the hip and other joints. The other is the end-effector type system in which the user's feet are placed on footplates that are moved using a manipulator.

With regard to the treadmill type, Hitachi Ltd. has commercialized rehabilitation systems using two belt type treadmills, which are already being used in many hospitals. However, the motion they generate is only in the horizontal direction. For exoskeletal type systems, LOPES [8] and HAL [9] have been developed. These are often used with a treadmill or a perambulator for gait rehabilitation. Colombo et al. have developed a gait training system with a treadmill and 4 DOF robots, which they have named Lokomat [10]. This system can be used to teach patients the correct motion; however, the equipment is complicated and cumbersome for both installing and removing the patient. As an end-effector type system, Hesse and Uhlenbrock have developed a gait trainer [11] and the Haptic Walker [12]. These systems have two footplates, which move the patient's feet. They have used the Haptic Walker to develop a training system for climbing stairs [13], as well as one for walking on flat surfaces. Currently, their system, which is named the “G-EO-System,” is commercialized by Reha Technologies [14]. Such systems help physiotherapists to reduce their manual labor and allow them time to provide quality support while helping patients get more exercise in anticipation of attaining a higher level of recovery. However, there are very few robotic gait rehabilitation systems for “in-house” gait rehabilitation, because the systems described above are too bulky to be brought into the home, and it is difficult for patients to attach themselves to and detach themselves from these devices.

Since 2000, the authors have been developing gait rehabilitation systems [15, 16] that are categorized as end-effecter type systems. These systems are equipped with two 2 DOF manipulators to move each of the patient's feet. Some of them have been used in hospital and have improved the gait abilities of stroke patients, such as their walking speed. However, these systems are also bulky for home use, even though the user can easily attach and detach themselves to and from the equipment. For home use, the system should be small. In this study a small end-effector type gait rehabilitation system has been developed. Its effectiveness was verified through experiments.

## 3. Gait Rehabilitation System for Home-Visit Rehabilitation

### 3.1. General Concept

For this study, we assumed that the gait rehabilitation system would be used during home-visit rehabilitation for patients who have suffered a stroke, can walk unattended, and have a low risk of heart disease. Independent walking can be achieved through the use of a weight-bearing device. After discharge from hospital, they return home and undergo rehabilitation. Since they have no robotic system at home, a physical therapist goes to the patient's home carrying the system in the trunk of their car. The therapist brings the system into the house and sets it up, and then the patient undergoes gait rehabilitation with the system. After the session, the therapist packs up the system and returns to the hospital.

At this time, the requirements of the system are considered to be as follows.

(R1) Repetition of the walking motion: the gait and balance should be stored in memory and repetition of the correct walking motion is required.
(R2)  Training of the vastus medialis muscle: to reduce the risk of falling, the performances of the eccentric and concentric contractions of the vastus medialis muscle are important. Eccentric contraction is usually used in descending a slope or staircase, and concentric contraction occurs when climbing a slope or staircase. The capability to train the vastus medialis muscle is an important aspect for home-visit rehabilitation.
(R3) Compact and small size: the system should not occupy a large space, since this cannot be expected in a normal household. In order for it to be carried by car, the size of the system should be minimized so that it is smaller than an ordinary suitcase.
(R4)  Lightweight: the system should be sufficiently lightweight to be carried by one person.
(R5) The devices that restrain the foot should be easy to attach and detach.

Systems that, potentially, satisfy the above requirements, are exoskeletal robots, such as HAL, and end-effector type systems, such as the Hapticwalker and the GaitMaster. However, to satisfy the requirement (R5), current exoskeletal robot type systems are unsuitable, because the rehabilitation is undergone within a restricted time frame (e.g., a 40-minute session) and attaching and detaching the patient to and from the equipment is too time-consuming for both the patient and the therapist.

The end-effector type gait rehabilitation systems can, potentially, also satisfy all the requirements. Unfortunately, none of the current end effector type systems are sufficiently compact. In addition, rendering algorithms for walking on slopes have not yet been developed.

In addition to the above, the device should pay attention to safety measures and should be a customer-friendly device for the physical therapist. Of course, the cost should also be considered. Thus, there are many aspects that need to be considered from the standpoint of practical applications.

In this feasibility study, a prototype compact gait rehabilitation system that satisfies the requirements (R1)–(R5) was proposed and developed, and a ramp-walking program was implemented to satisfy requirement (R2).

### 3.2. Hardware Design

**Figure 1:**
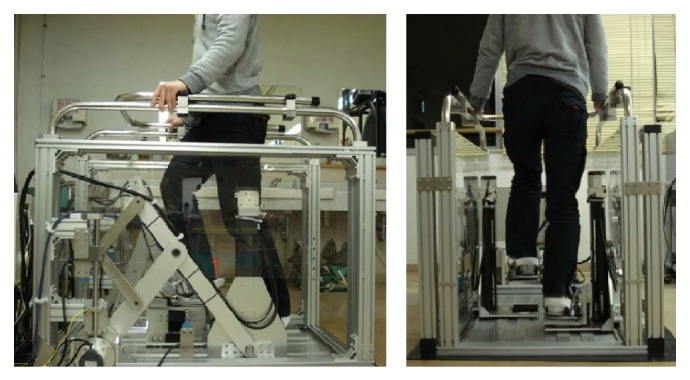
System overview of the GM5.

In previous work, we developed the GM5, which consists of two 2 DOF manipulators equipped with footpads (Figure 1). The design of the GM5 was undertaken with the following constraints in mind.

- The patient is able to do repetitive exercise and hip extension and flexion exercises following the movements of the feet of a healthy individual when walking.
- Each foot can be fastened to a footpad.

Considering the average size of the patient, we have the following.

- The footpads can move 700 mm horizontally and 315 mm vertically.
- The maximum walking speed is 1000 mm/s and the rated payload of each motion-platform is approximately 80 kg.
- It uses a 100 V power supply, which is common in Japan, for cost reduction.

For this new study, the requirements outlined above were discussed with physical therapists familiar with our previous systems. Since it is difficult to satisfy all of the requirements, these were prioritized in these discussions. As a result, the highest priority was given to realizing the walking motion of the feet, even if the range of movement had to be reduced, because repetitive exercise and hip extension and flexion are important factors in maintaining and developing walking ability. Therefore, taking into account requirement (R3), the horizontal and vertical ranges of movement of the system were reduced from those used in the GM5 to 300 mm horizontally and 300 mm vertically. The user can walk on a level surface or a slope with a 300 mm step length. In addition, the maximum walking speed was reduced to 500 mm/s in order to reduce cost. These features also led to a reduction in the drive noise of the device.

Considering the requirement (R2), a real slope is inclined, so the footpads need to be also. However, an active mechanism to achieve this and a method to control such a mechanism are complicated and would make the equipment bulky and high cost.

Based on the above consideration, the design constraints of new system were determined to be as follows:

(R-1) the capability of generating motion for the user's feet following the prerecorded movement of a healthy individual walking;
(R-2) the user can walk on a virtual slope;
(R-3) only the feet are fastened to footpads;
(R-4) the footpads can move 300 mm vertically and 300 mm horizontally;
(R-5) the rated walking speed is 500 mm/s and the rated payload of each motion-platform is approximately 80 kg;
(R-6) it uses a 100 V power supply.

## 4. Prototype System

### 4.1. Hardware of the System

**Figure 2:**
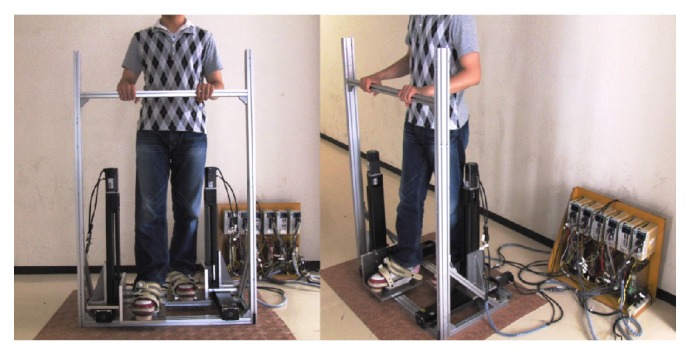
Mechanism of the footpad of the GMmini.

A prototype system named the GaitMasterMini (GMmini for short) was developed in this study. The GMmini consists of two 2 DOF motion-platforms (Figure 2) and a PC for control.

**Figure 3:**
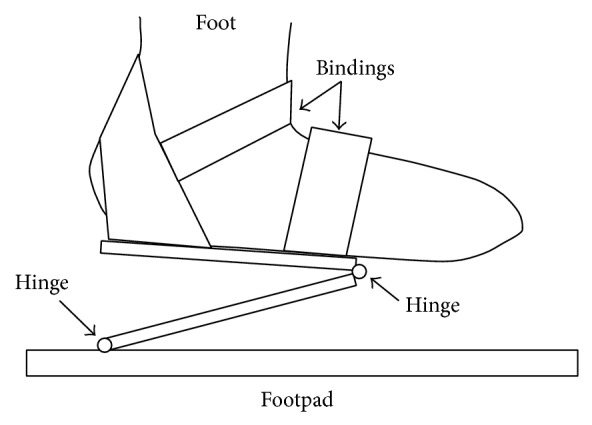
Mechanism of the footpad of the GMmini.

Each motion-platform has two linear actuators placed perpendicular to each other. One is for the back-and-forth motion and the other is for the up-and-down motion. Each actuator comprises a linear slider, KR45H10A+440L (made by THK), and an AC servomotor, HC-KFS43 (made by Mitsubishi Electric Company). They have a movable range of 315 mm. The footpads can move at a speed of 520 mm/s in both the horizontal and vertical directions. Each motor is equipped with a rotary shaft encoder, with which the position of each footpad can be measured. The size of the motion-platform system is 1380 mm (*H*) × 860 mm (*W*) × 560 mm (*D*). Thus, the footprint of the GMmini is 0.5 m^2^, compared with 2.3 m^2^ for the GM4 and 1.1 m^2^ for the GM5. The size of the footpad is 270 mm (*W*) × 300 mm (*D*). To fix the user's feet on the footpads, we use the bindings used for a snowboard (Figure 3). The foot joints are fixed in such a manner that the toes and heels can move freely, so that the dorsal flexion and plantar flexion of the foot joints are free. The system is equipped with a safety bar placed in front of the patient. The footpads and safety bar of the system can safely support the patient's body. The mass of this system is about 70 kg. Compared with the previous GM systems, the width, depth, and mass have been decreased, and it has a simple structure that is easy to assemble and disassemble.

On the PC, an encoder counter board (PCI-6205C, made by Interface Corp.) and a digital-to-analog converter board (PCI-3341A, made by Interface Corp.) are installed. Each motor is driven by a driver (MR-J2S-40A1, made by Mitsubishi Electric Company) used in the angular velocity control mode. When the PC receives the angle data from the encoder, it calculates the position of each footpad. The PC calculates the difference between the destination position and the current position and then outputs angular velocity data proportional to the difference. The destination position is set based on a trajectory captured from a healthy individual. The size and circulation time of the trajectory are modified taking into account the body size and condition of the user. The trajectory of each footpad can easily be changed by changing the motion data.

### 4.2. Gait Rehabilitation Program

Gait rehabilitation programs for walking on flat surfaces, and ascending and descending stairs have been developed using previous GMs [5]. In these modes, the trajectories of the footpads were generated using the motion data of the ankle of a healthy individual. The motions were prerecorded using a motion capture system (Stereo Labeling Camera, made by CyVerse). In the swing phase, the footpads were moved according to the prerecorded absolute positions of the feet. In the stance phase, they were moved backwards.

In a preliminary experiment, walking on a real slope was observed. A healthy individual walked on a treadmill that could be tilted from 0 to 15 degrees. The position data of the ankle was captured for every 5-degree angle of tilt.

**Figure 4:**
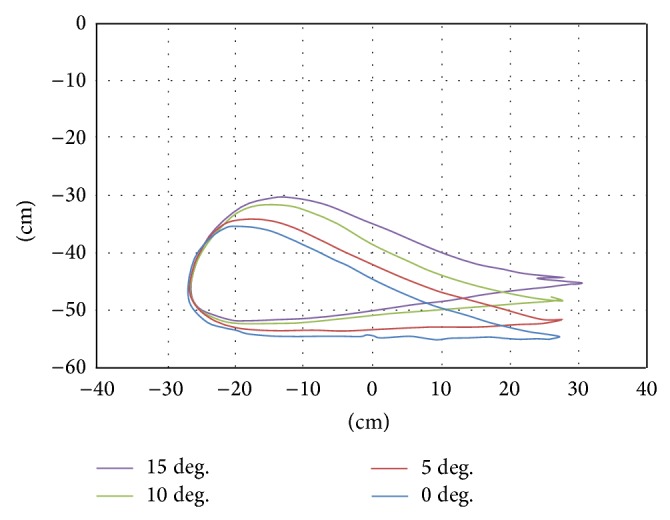
Trajectories of foot during walking on real slopes.

**Figure 5:**
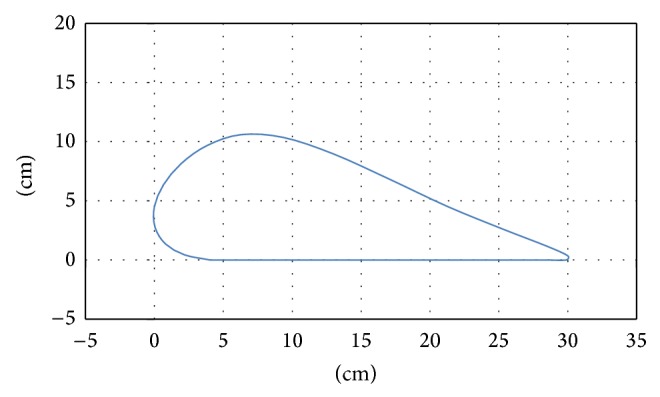
Trajectory of the footpad in walking on a flat surface.

The results showed the walking trajectories on a slope to be the same as those on a flat surface but with an added tilt (Figure 4). Therefore, we developed the trajectory for walking on a slope by rotating the trajectory used for walking on a flat surface (Figure 5).

**Figure 6:**
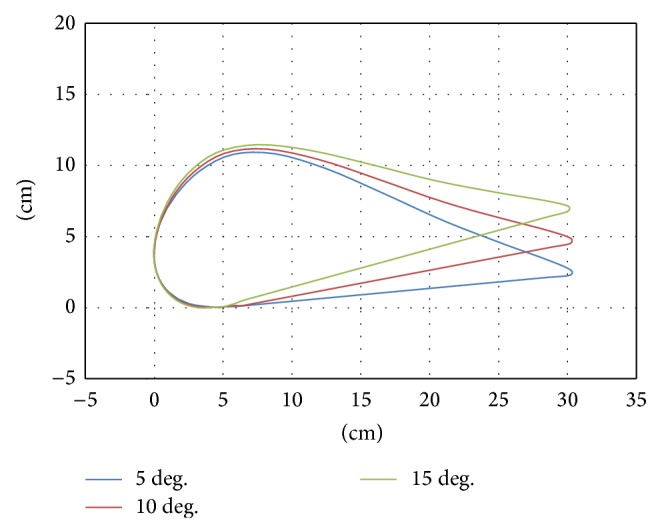
Trajectory of the footpad in acclivity walking.

**Figure 7:**
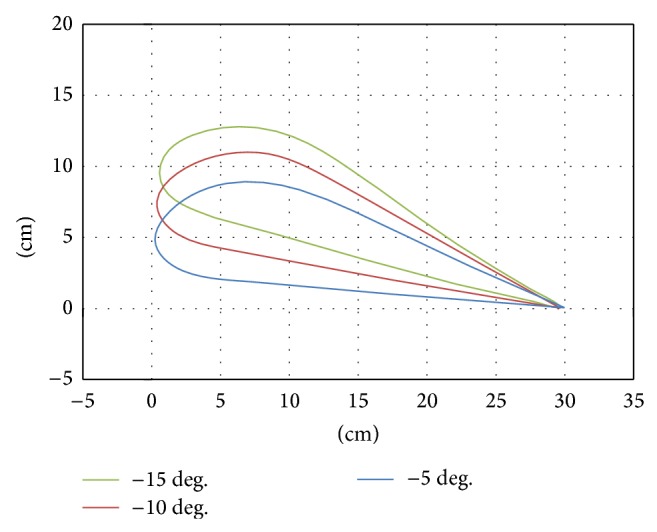
Trajectory of the footpad in declivity walking.

A combination of rotational transformation and vertical translation matrices (Equation (1)), was used to rotate the trajectory data, where *x*′′ and *y*′′ refer to the coordinates of the tilted trajectory, *x* and *y* are the coordinates of points for walking on a flat surface, *α* is the tilt angle, and *y*′ is the minimum value of *y* after tilting. The minimum point of the tilted trajectory was often underneath the floor. In order to set the height of the lowest point to zero (*y* = 0), the tilted *y* was subtracted from the *y*′ value after tilting (Figures 6 and 7). Consider

(1) $$\begin{array}{c} \left[\begin{array}{c} x^{\prime \prime }\\ y^{\prime \prime } \end{array}\right]=\left[\begin{array}{cc} \cos\alpha & -\sin\alpha \\ \sin\alpha & \cos\alpha \end{array}\right]\left[\begin{array}{c} x\\ y \end{array}\right]-\left[\begin{array}{c} 0\\ y^{\prime } \end{array}\right]. \end{array}$$

Before developing the GMmini, we tested the slope-rendering algorithm on the GM4, which is a previous version of the GaitMaster. Since the GM4 has no inclination mechanism, the angle between the sole of the foot and the floor is different from the angle between the sole of the foot and the floor on a real slope. Some users, however, reported that they felt as if they were walking on an actual slope. This indicates that the inclination mechanism might be unnecessary in the system and that the cost of the device can be kept down. Therefore, the GMmini was developed without an active inclination mechanism.

In addition, the step length of the slope trajectory was limited to 300 mm in order to downsize the GM in this study.

## 5. Evaluation of Climbing and Descending Slopes with the System

To evaluate the system for gait rehabilitation in the home, we compared walking on real slopes with walking on the GMmini. In addition, since the footpads have no active inclination mechanism, the influence of a passive incline was tested in this experiment.

### 5.1. Experimental Setup

**Figure 8:**
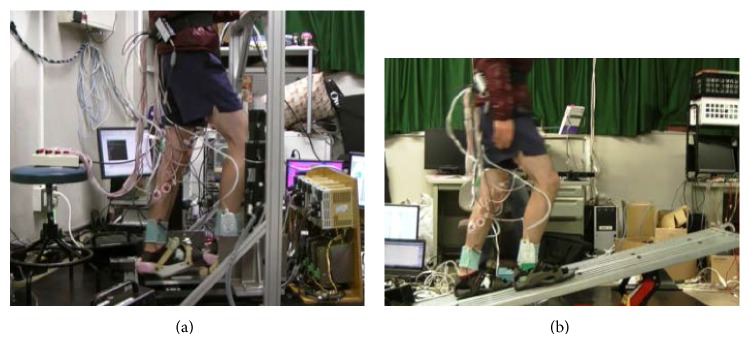
Experiment environment ((a) on GMmini and (b) on a real slope).

In this experiment, the subjects walked on real slopes made from a 1.5 m long steel board (Figure 8(b)) and on virtual slopes generated by the GMmini (Figure 8(a)). To generate virtual slopes, six trajectories for walking on ramps with inclination angles of 5, 10, and 15 degrees (Figure 6), and −5, −10, and −15 degrees (Figure 7) were used. The real slopes had the same inclination angles as the virtual slopes.

**Figure 9:**
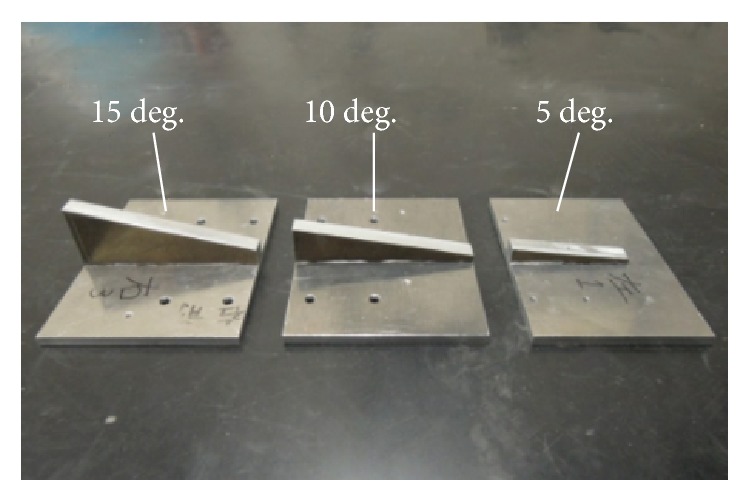
Three wedges for inclination of the footpad.

Also, to evaluate the inclination of the soles of the subjects' feet on the footpads, 3 sets of wedges (Figure 9) with 5, 10, and 15-degree angles were prepared. By placing a wedge between each footpad and the foot, the sole could be inclined from −15 degrees to 15 degrees during the stance phase.

The subjects walked for 20 steps under each condition. On the real slope, the subjects walked from one end of the steel board to the other and then returned to the initial position. This was repeated 4 times for each real slope condition.

In this experiment, the walking speed was set to 0.42 m/s with an average cadence of 1.4 steps/s calculated from data obtained from actual gait rehabilitation exercises done in 2007 to 2008 using the GM4. On the real slope, the subjects walked to the sound of a metronome. During the experiment, electromyograms of the subjects, their motion, and the inclination of their ankles were recorded.

An electromyograph (EMG), the MEG-6108 manufactured by Nihon Kohden, which has 16 EMG channels, was used for measuring the muscle activity of the subjects. The electromyograms of four types of muscle were measured in this experiment. These were the gluteus medius muscle, the hamstring, the vastus medialis muscle, and the gastrocnemius muscle. The gluteus medius muscle is a muscle of the outer surface of the ilium. This muscle is in charge of rotation of the thigh. The muscle pulls the thigh away from midline and supports the body on one leg to prevent the pelvis from dropping to the opposite side. The hamstring is the muscle at the back of the thigh and controls the extension of the hip joint and the flexion of the knee joint. It decelerates the lower limb of the swinging leg when switching from the swing phase to the stance phase. The vastus medialis muscle is a knee muscle, which absorbs the shock when the foot lands on the ground. The gastrocnemius muscle is a calf muscle that controls the flexion of the knee joint and the springing motion used for acceleration [17].

**Figure 10:**
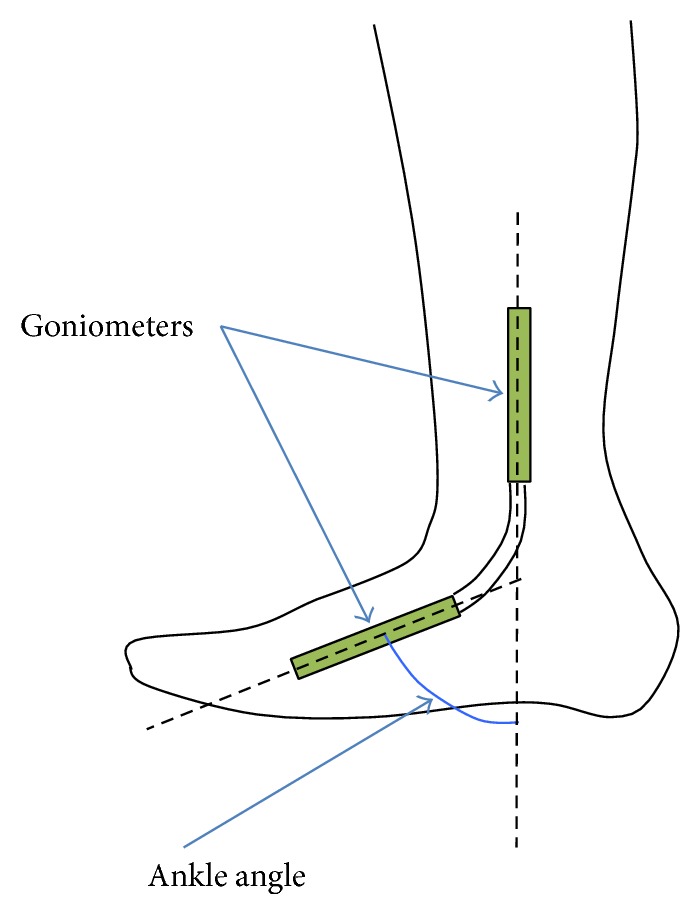
Goniometer attached to the ankle of a subject.

To measure the inclination of the subject's ankle, a goniometer (SG150, made by Biometrics Ltd) was attached to the subject's ankle (Figure 10). In this experiment, the angle of the ankle is defined as shown in Figure 10.

Six healthy individuals in their 20s participated in this experiment. Each took part in three time trials conducted under each condition.

### 5.2. Video Analysis

**Figure 11:**
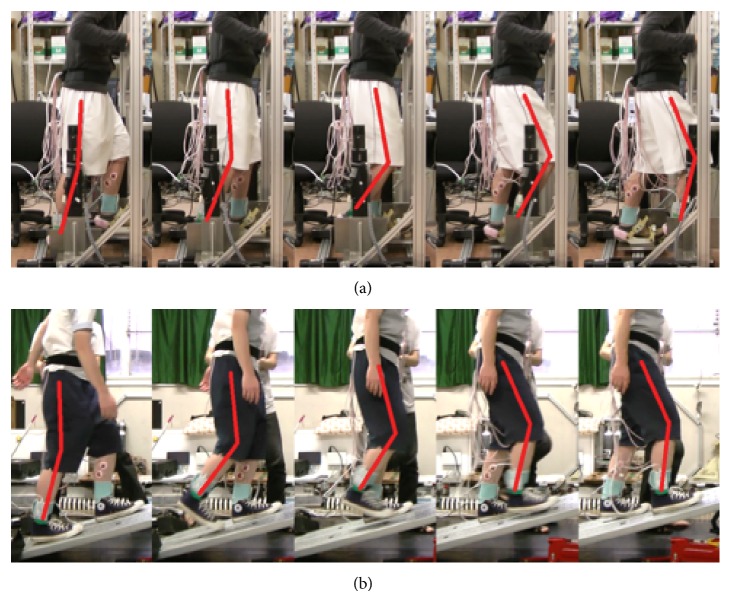
Typical motion during slope climbing ((a) on the GMmini and (b) on a real slope).

**Figure 12:**
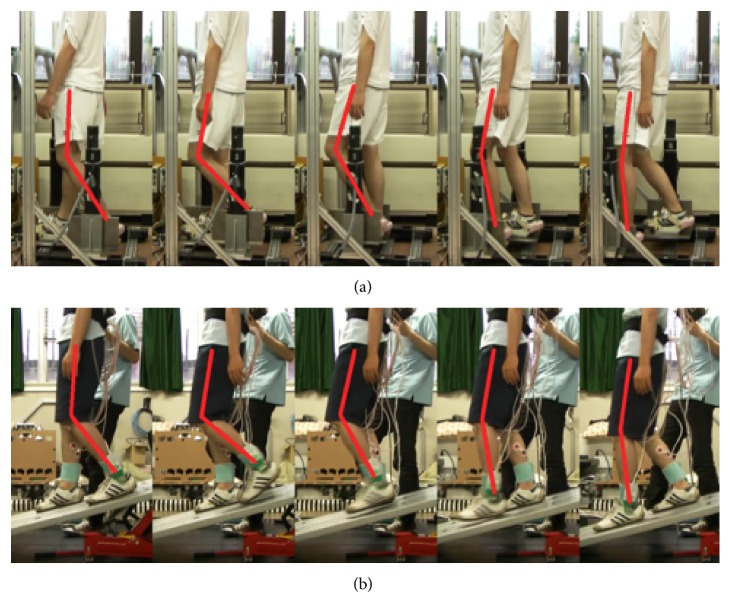
Typical motion during slope descending ((a) on the GMmini and (b) on a real slope).

Figures 11 and 12 show the typical motions used in ascending and descending slopes. The top of each figure shows the subject's motion on the GMmini, and the bottom of each figure shows the subject's motion on the real slope. To aid understanding of the motion, the positions of bones are highlighted in red.

From Figure 11, the positions of the lower extremity on the GMmini are the same as those on the real slope in each swing phase. At the end of the stance phase (the second figures from the left of Figure 11), however, the subject on the real slope tended to flex his knee joint more than on the GMmini, in order to prevent his toe colliding with the slope, whereas, on the GMmin the subject's toe was fixed to the footpad and there was no danger of a collision.

From Figure 12, the motion of the knee and thigh on the GMmini resembles that on the real slope. At the end of the stance phase (the second figures from the left of Figure 12), however, the subject on the real slope tended to flex his knee more than on the GMmini, because of wanting to avoid his toe colliding with the slope.

### 5.3. Angle of Ankle during Walking up a Ramp on the GMmini

**Figure 13:**
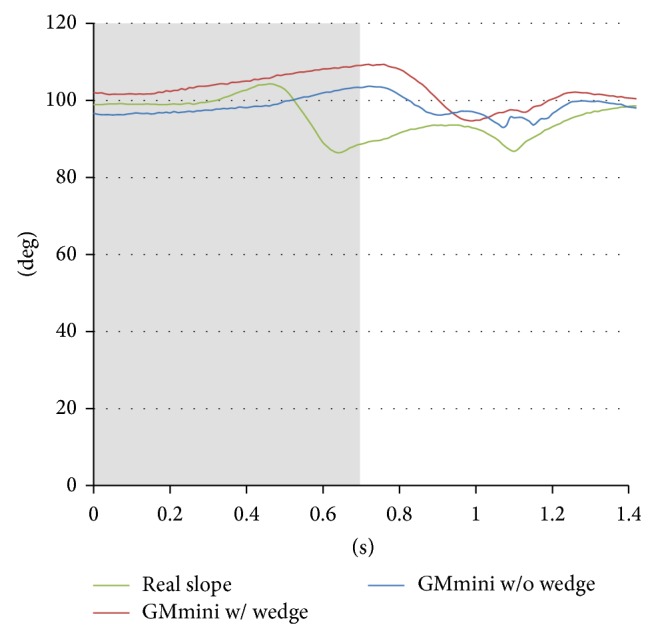
Time series variation of the average angle of the subjects' ankle during ascent of a 5-degree incline.

Time series variations of the angle of the subjects' ankles during climbing the slopes were measured with the goniometer. An example is shown in Figure 13, where the average angle of the subjects' ankles while ascending a slope with an inclination of 5 degrees was measured. The gray colored area in Figure 13 indicates the stance phase.

In the stance phase, the angle gradually increases with the backward motion of the footpad. In the GMmini with the wedge, since the sole of the foot was in continual contact with the footpad, the angle is larger than that without the wedge. This means that the subjects tended to keep their heels on the footpad even during the swing phase. On the other hand, when walking on a real slope the feet can move freely, so that the angle of the ankle decreases quickly at the end of the stance phase, because the subjects tended to put their toes on the slope, while the heels began to move upward. Also just prior to landing on the slope, the ankle was extended in order to provide a soft-landing.

**Figure 14:**
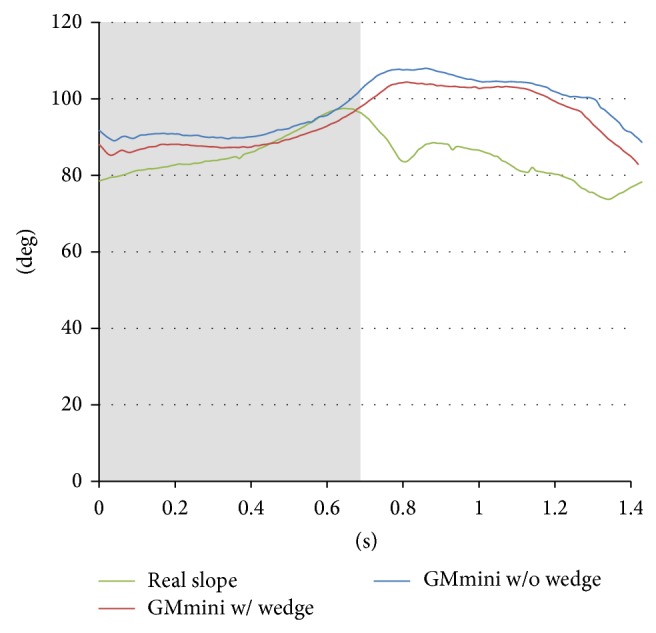
Time series variation of the average angle of the subjects' ankle during descent of a 5-degree incline.

Figure 14 shows the time series variation of the average angle of the subjects' ankles during descent of a 5-degree incline. In the stance phase, the angle gradually increases in all cases. In the swing phase on the GMmini without a wedge, since the sole of the foot is continually in contact with the footpad, the angle is larger than that with the wedge. This means that the subjects tended to put their heels on the footpad in the swing phase. On the other hand, the angle of the ankle in the swing phase when walking on the real slope gradually decreases in order to prepare for a soft-landing.

These results show that the angle of the ankle on the real slope is different to that on the GMmini. However, the motion of the ankle on the GMmini is almost the same as that on a real slope, because the trajectory of the footpad was derived from data captured from the motion of the ankle of a healthy individual on a real slope. Therefore, some users on the GMmini felt that they were walking on a real slope even though the angle of the ankle was different.

### 5.4. EMG Analysis

**Figure 15:**
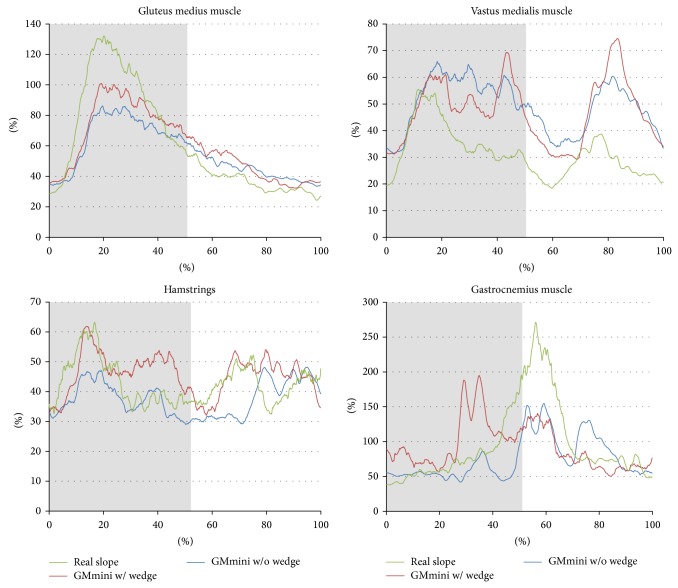
Time series variation of the average RMS values of the EMGs during ascent of a 15-degree incline.

The muscle function was measured with EMGs to evaluate the effect of training to climb slopes.  Figure 15 shows the time series variations of the normalized root mean square (RMS) values of the average electromyograms of the leg muscles of the six healthy subjects ascending slopes. The data points for each muscle under each condition were calculated using the following procedure. (1) The RMS values were obtained from the time series variations of the EMG raw data smoothed by a simple moving average of the previous 120 data points. (2) These were normalized by the subject's maximum voluntary contraction in climbing a 5 degree (for uphill)/−5 degree (for downhill) slope without a wedge on the GMmini. (3) The RMS values in each time slot were normalized over each stride to convert it within 0 to 100%. (4) The average RMS values were calculated using all the subjects' RMS values.

Figure 15 shows the average RMS values for each condition while climbing a 15-degree slope. As a result, although the intensities of the RMS values for each muscle are different from each other, the profiles are almost same. From the viewpoint of intensity, the muscle activities of the gluteus medius and the vastus medialis muscles on the real slope are different to those on the GMmini.

The activity of the vastus medialis muscle when using the GMmini is greater than that on the real slope. At the beginning of the stance phase in all conditions, concentric contraction of the muscle occurred in order to lift the subject's body revealed from the analysis of the EMG and the video. However, after contraction, the activity of the muscle was maintained when using the GMmini. Since the footpad moved the foot back in the stance phase, the stiffness in the knee joint increased continuously to avoid falling. A peak occurs in the swing phase when using the GMmini. This is caused by the extension of the knee joint before the heel makes contact. Since each foot was constrained by being attached to a footpad and the footpad had an action similar to wearing heavy shoe, each muscle needed to be more active than when climbing a real slope before the heel makes contact.

For the gastrocnemius muscle, a large peak appears at the beginning of the swing phase on the real slope, while these peaks are smaller on the GMmimi. On the real slope, the subjects should lift their bodies by pressing down on the slope. By contrast, the subjects were moderately restrained by the footpads on the GMmini, and, in addition, held on to the safety bar. This caused a lower intensity in the activity of the gastrocnemius muscle on the GMmini than that on the real slope. Holding on to the safety bar also caused lower activation of the gluteus medius muscle when using the GMmini.

**Figure 16:**
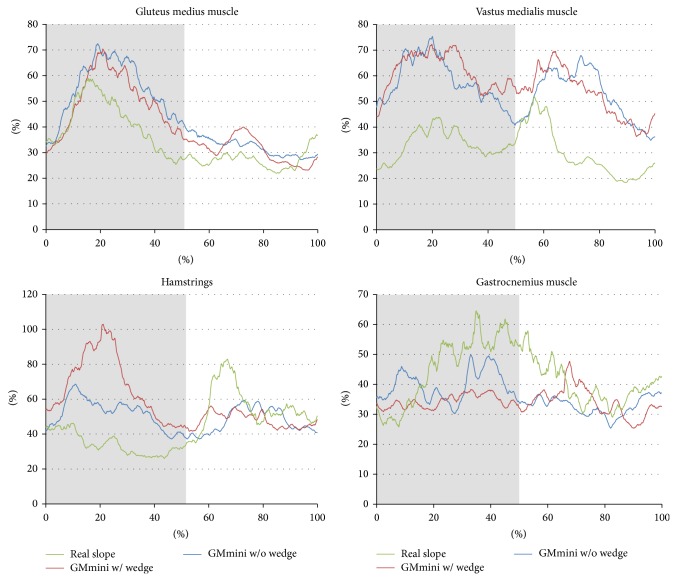
Time series variation of the average RMS values of the EMGs during descent a −15-degree incline.

Figure 16 shows the average RMS values for each condition during descent of a −15 degree slope. The results show the profiles of the RMS values to be almost the same regardless of the condition. However, as with ascending, there is a dependency on condition for the intensities of the muscle activities of the vastus medialis and gastrocnemius muscles. In the stance phase, eccentric contraction of the vastus medialis muscle to let the body down was observed in all conditions revealed from the analysis of the EMG and the video. When using the GMmini, the moving footpads caused the muscle activity to have a greater intensity than that when on the real slope. In addition, with the wedge, the hamstrings, which are an antagonist of the vastus medialis muscle, were also active. The reason for this is that the subjects tended to stiffen the knee joint during the stance phase when descending the slope in order to prevent falling. A peak in the muscle activity of the vastus medialis muscle was found in the swing phase, in all conditions. This peak is due to extension of the knee joint. When using the GMmini, the duration of the peak was longer than on the real slope. The subjects tended to put their weight on the footpad in the swing phase, and, in order to follow the motion of the footpad, the vastus medialis muscle became more active. Grasping the safety bar also caused lower activation of the other muscles when using the GMmini.

**Figure 17:**
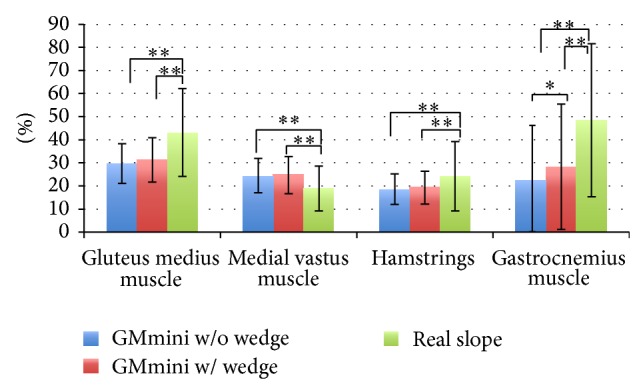
Average muscle activities while ascending a slope.

Figure 17 shows the results of a comparison of the average muscle activity of healthy subjects during ascent of a slope. The average muscle activity was calculated by averaging the normalized RMS values over each stride. The error bars indicate the standard deviation in each case. Two-way ANOVA found a statistically significant difference in each muscle. Post hoc analysis using Scheffe's Multiple Comparison test reveals significant differences for each muscle. ∗∗ refers to the 1% significance level and ∗ refers to the 5% significance level. The effect of each muscle is shown in Table 1. The figure shows the same tendencies shown in Figure 15.

Moreover, comparison of the activities when using the GMmini with and without a wedge shows no significant difference for all muscles except the gastrocnemius muscle. This is because the subjects had to generate more force using the gastrocnemius muscle to lift their bodies when the floor was inclined. However, the activities of the other muscles were at about the same level. In particular, the activity of the vastus medialis muscle increased compared with that on the real slope.

**Figure 18:**
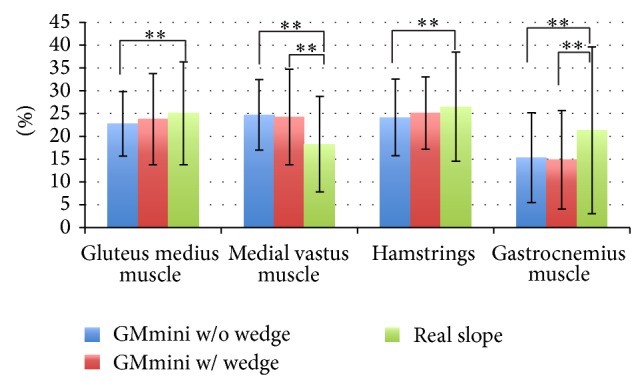
Average muscle activities while descending a slope.

Figure 18 shows the results of a comparison of the average muscle activity of the healthy subjects during descent of a slope. Two-way ANOVA found a statistically significant difference in each muscle. Post hoc analysis using Scheffe's Multiple Comparison test reveals significant differences in each muscle. The effect of each muscle is shown in Table 2. The figure shows the same tendencies shown in Figure 16. Comparison of the activities when using the GMmini with and without a wedge shows no significant difference for all muscles. The activity of the vastus medialis muscle in particular has increased compared with that on the real slope.

These results demonstrate the effectiveness of the GMmini.

## 6. Discussion

In this study, the requirements of a gait rehabilitation system for home-visit rehabilitation were investigated. The major difference to related work is that the system needs to be compact. The GMmini is a compact and small gait rehabilitation system. It can provide uniform repetitive gait movement, constrained gait movement, pelvic rotation, and hip extension and flexion.

Although the step length on the GMmini is limited to 30 cm, the amount of exercise done when using the ramp-walking mode is comparable to that done on a natural slope. Moreover, the experimental results indicate that concentric and eccentric contractions of the vastus medialis muscle can be realized within the restricted time frame of home-visit rehabilitation. To increase the amount of exercise, a walking mode that gradually changes from ascending a slope to descending a slope and vice versa can be implemented, if needed. Moreover, by loosening his or her grip on the safety bar, the amount of exercise can also be increased when the patient has the ability and the balance required for walking.

From the result of the comparison with “GMmini with the wedge” and “GMmini without the wedge” shows no significant difference, except for the gastrocnemius muscle in the climbing the slope. This suggests that there is no need to implement the inclination mechanism on the footpad. To train the gastrocnemius muscle, the GMmini might provide effective ways. In this case, an exercise such as a squat exercise is effective to train the gastrocnemius muscle. The authors think that it is not necessary to follow all exercises with only the GMmini. Furthermore, this result suggests that managing the motion of the heel like a real walking is essential to generate the feeling of walking.

The prototype system weighs 70 kg and can be divided into several parts. Therefore, it is possible that each part can be carried individually. In addition, it is also possible to design a folding type of GMmini that can be stored in a large suitcase.

## 7. Conclusion

A compact and small gait rehabilitation system for home-visit rehabilitation was developed for this study. The system can be used for gait rehabilitation in the patient's home without the physical therapist becoming fatigued. Using a ramp-walking program, the patient can undergo rehabilitation that realizes hip extension and flexion and exercises the vastus medialis muscles, which are important to reduce the risk of falling. The effectiveness of the system was verified through an evaluation using 6 healthy individuals.

For future work, the protocols for rehabilitation with the system should be investigated. Indeed, the amount of exercise done with ramp walking is greater than that done walking on a flat surface. It is hard for a patient to walk for 20 minutes, which is the time spent walking during rehabilitation using the GaitMaster systems currently used in hospitals. It is necessary to find the optimal amount of exercise for each patient. Reducing the cost is another issue that needs to be addressed. We plan to develop a compact gait rehabilitation system with local companies to be put into practical use.

By using compact systems, patients and physical therapists can both be helped, thus increasing the quality of their lives.

## Figures and Tables

**Table 1:** Effect size of each muscle in slope climbing.

Muscle	*F*(2,1071)	*P*	Effect size
Gluteus medius	111.600	0.000 < .01	0.457
Vastus medialis	54.636	0.000 < .01	0.319
Hamstrings	31.974	0.000 < .01	0.244
Gastrocnemius	83.238	0.000 < .01	0.394

**Table 2:** Effect size of each muscle in slope descending.

Muscle	*F*(2,1071)	*P*	Effect size
Gluteus medius	5.393	0.005 < .01	0.100
Vastus medialis	50.842	0.000 < .01	0.308
Hamstrings	5.544	0.004 < .01	0.102
Gastrocnemius	25.796	0.000 < .01	0.219
